# pH-responsive delivery vehicle based on RGD-modified polydopamine-paclitaxel-loaded poly (3-hydroxybutyrate-co-3-hydroxyvalerate) nanoparticles for targeted therapy in hepatocellular carcinoma

**DOI:** 10.1186/s12951-021-00783-x

**Published:** 2021-02-06

**Authors:** Mingfang Wu, Chen Zhong, Qian Zhang, Lu Wang, Lingling Wang, Yanjie Liu, Xiaoxue Zhang, Xiuhua Zhao

**Affiliations:** 1 College of Chemistry, Chemical Engineering and Resource Utilization, Northeast Forestry University, 26 hexing road, Harbin, 150040 Heilongjiang China; 2 School of Biological and Chemical Engineering, Zhejiang University of Science and Technology, Hangzhou, 310023 Zhejiang China; 3 Key Laboratory of Forest Plant Ecology, Northeast Forestry University, Ministry of Education, Harbin, 150040 Heilongjiang China; 4 State Key Laboratory of Genetic Engineering, School of Life Sciences, Fudan University, Shanghai, 200438 China

**Keywords:** PHBV, Dopamine, RGD peptide, pH-sensitive, Active targeting

## Abstract

A limitation of current anticancer nanocarriers is the contradiction between multiple functions and favorable biocompatibility. Thus, we aimed to develop a compatible drug delivery system loaded with paclitaxel (PTX) for hepatocellular carcinoma (HCC) therapy. A basic backbone, PTX-loaded poly (3-hydroxybutyrate-co-3-hydroxyvalerate) PHBV nanoparticle (PHBV-PTX-NPs), was prepared by emulsion solvent evaporation. As a gatekeeper, the pH-sensitive coating was formed by self-polymerization of dopamine (PDA). The HCC-targeted arginine-glycine-aspartic acid (RGD)-peptide and PDA-coated nanoparticles (NPs) were combined through the Michael addition. Subsequently, the physicochemical properties of RGD-PDA-PHBV-PTX-NPs were characterized by dynamic light scattering-autosizer, transmission electron microscope, fourier transform infrared spectroscopy, differential scanning calorimetry, thermogravimetry and X-ray spectroscopy. As expected, the RGD-PDA-PHBV-PTX-NPs showed robust anticancer efficacy in a xenograft mouse model. More importantly, they exhibited lower toxicity than PTX to normal hepatocytes and mouse in vitro and in vivo, respectively. Taken together, these results indicate that the RGD-PDA-PHBV-PTX-NPs are potentially beneficial for easing conflict between multifunction and biocompatible characters of nanocarriers. 
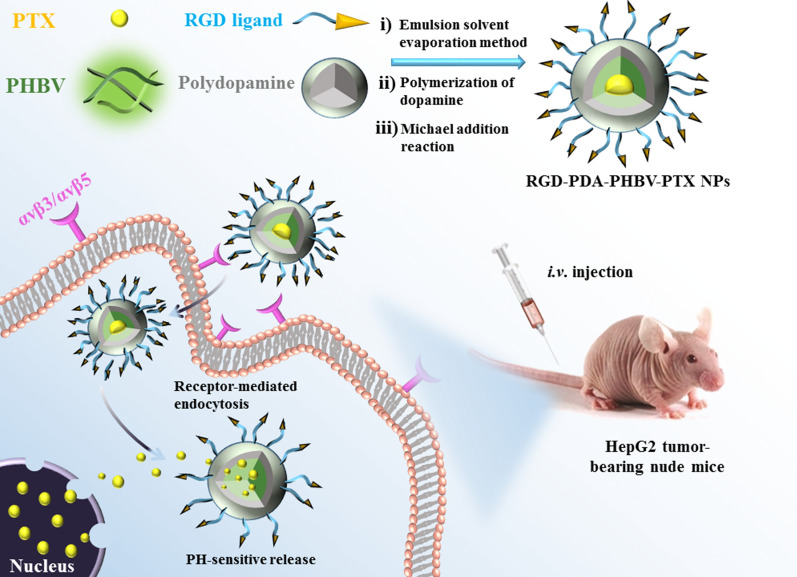

## Introduction

Hepatocellular carcinoma (HCC) is one of the leading causes of cancer death worldwide [1]. Especially, in developing countries, the incidence rate of HCC increased significantly year by year. The pathogenesis of HCC is strongly correlated with chronic infection of hepatitis B virus, hepatitis C virus, alcoholic liver cirrhosis, etc. [2, 3]. Conventional treatments, for patients at early stages of HCC, include surgery, radiotherapy, interventional therapy and radiofrequency ablation [4]. Although surgical resection is the first choice for patients, the recurrence rate can reach more than 30% within 5 years after operation [5]. In addition, most HCC patients are diagnosed at an advanced stage when curative therapies such as surgical resection and liver transplantation are commonly inapplicable. In this situation, systemic chemotherapy remains the mainstream treatment for HCC patients [6].

Paclitaxel (PTX), a diterpenoid compound, isolated from the needle biomass of yew tree *Taxus baccata*, has been developed a natural broad-spectrum antineoplastic drug due to its potent anticancer activity [7]. PTX is capable of promoting tubulin polymerization, thereby resulting in abnormal arrangement of vascular bundles, and eventually leading to the death of cancer cells [8, 9]. However, its low water solubility and serious side effects bring great challenges to clinical drug administration [10, 11]. Therefore, there is an urgent need to develop an effective method to solve the existing shortcomings of PTX. As the development of nanotechnology and the in-depth study of tumor microenvironment, nanoparticle drug delivery system has attracted significant attention [12]. The traditional administration of chemotherapeutics is intravenous injection. The drugs are rapidly distributed throughout the body through the blood circulation [13, 14]. It generally results in a short blood half-life and prominent off-target accumulation in multiple healthy organs. This, together with the large volume of administration of chemotherapeutic agents and their unspecific mechanism of action, causes severe side effects [15]. Therefore, the novel chemotherapeutic agents or strategies are urgently needed to treat HCC patients with minimal or tolerable side effects [16].

More recently, the use of biodegradable nanomaterials has received impressive attention. Ploy (3-hydroxybutyrate-co-3-hydroxyvalerate) (PHBV), a biodegradable biopolymer with a low glass transition temperature and a hard texture, which is produced by using starch as raw material and fermentation engineering technology [16, 17]. PHBV has a variety of advantages such as favorable biocompatibility and low manufacturing cost. Thus, it is a suitable biomaterial for the controlled release drug delivery systems [18, 19]. To date, a number of studies reported that the drug tightness can be improved by loading with polymer carriers. Some macromoleclar polymers, such as poly lactic-co-glycolic acid (PLGA), polylactide (PLA) and poly-caprolactone (PCL), have been used as the carriers (Additional file 1: Table S1). Compared with other attractive polymers such as PLGA, PLC and PLA, PHBV possesses lower manufacturing cost and longer entrapment time to prevent premature drug leakage [20]. For example, Fatemeh et al. [21] prepared PLGA-PVA-PTX NPs with the particle size of 223 nm, whilst 88.3% of PTX was released into the buffer solution (pH = 7.4) within 120 h. In contrast, Cristian et al. [22] prepared PTX NPs based on the PHBV. The NPs exhibited better sustained-release performance because less than 1% of PTX was liberated in the physiological solution (pH = 7.4) over a 5-day period. The outer surface of nanoparticles (NPs) is often modified by various ligands that interact with cells to improve tumor targeting and uptake [23]. Arginine-glycine-aspartic acid (RGD) peptide can specifically bind to integrins on tumor cells, thereby achieving tumor-targeting effect [24, 25]. Due to the lack of specific functional groups on the surface of NPs, RGD cannot directly couple with NPs. Under the weak alkaline condition (pH-8 to -8.5), the catechol of dopamine is oxidized to quinone, and then reacts with other catechols or quinones to form polymer films [26]. Therefore, the polydopamine (PDA)-modified NP surface is able to combine functional ligands such as peptides, nucleic acids and small molecules [27].

The purpose of this study is to establish a compatible NPs-based drug delivery system, which has the characteristics of pH responsiveness, tumor targeting as well as biodegradation. First of all, the PTX was loaded with PHBV to construct the basic drug delivery carrier. Secondly, the thin film formed by the self-polymerization of dopamine (PDA) was used as the shell of pH response switch. Finally, the coupling ligand RGD was grafted to the PDA to form the complete NPs. To our best knowledge, the use of PHBV as carrier for targeted therapy of HCC has not been reported yet. In current study, we have carried out a comprehensive process optimization for the preparation of PHBV-PTX-NPs. It provides a stable basis for subsequent functional preparation. In addition, the characterization, morphology, stability, release, uptake, biodistribution and anticancer efficacy of functionalized NPs were evaluated. Our data indicate that RGD-PDA-PHBV-PTX-NPs are effective for HCC treatment both in vitro and in vivo experiments. This is attributed to the abilities of RGD-PDA-PHBV-PTX-NPs in the specific response to acidic tumor microenvironment, the sustained PTX release and the targeted property for HCC cells.

## Materials and methods

### Materials

PHBV (PHV content 12 mol %), PTX (purity 98%), hydrochloride dopamine, polyethylene glycol, 3-(4,5-dimethylthiazol-2-yl)-2,5-diphenyl-2 H-tetrazolium bromide (MTT) and diamidino-2-phenylindole (DAPI) were purchased from Sigma-Aldrich (St. Louis, MO, USA). Arginine-glycine-aspartic acid (RGD) was bought from Dalian Meilun Biology Technology Co., Ltd. (Dalian, China). High-Performance Liquid Chromatography (HPLC)-grade methanol and acetonitrile were purchased from Mallinckrodt Baker (Phillipsburg, NJ, USA). Fluorescein isothiocyanate (FITC) and 1,1′-dioctadecyl-3,3,3′,3′-tetramethylindotricarbocyanine iodide (DIR) were purchased from Invitrogen (Carlsbad, CA, USA). The HCC cell lines HepG2 and SMMC-7721, and human hepatocyte cell line HL-7702 were purchased from the Institute of Biochemistry Cell Biology (Shanghai, China).

### Preparation of PHBV-PTX-NPs

PHBV-PTX-NPs were prepared by emulsion solvent evaporation. Typically, PHBV (300 mg) and PTX (100 mg) were added to 10 mL of dichloromethane (referenced to pre-experiment) as oil phase (Additional file 1: Fig. S1), and 105 mg of polyvinyl alcohol (PVA) was dissolved in deionized water as water phase. The above two suspensions were dispersed for 10 min in Elmasonic TI-H-5 ultrasonic bath (Elma, Singen, Germany). The oil phase solution of dichloromethane was gradually injected into the aqueous solution containing PVA under the action of adjustable High-Speed Homogenizer Stirrer (Yineng Experimental Instrument Factory, Changzhou, China) to produce stable emulsion. Subsequently, the milky white nano-emulsion was formed in the homogenization-pressure nano-homogenizer (ATS Engineering, Shanghai, China). The PHBV-PTX-NPs suspension without organic solvents was obtained by rotating evaporation. The PHBV-PTX-NPs were centrifuged at 10,000 rpm for 10 min and washed three times in 10 mL of deionized water to remove unencapsulated drug and PVA emulsifier. The PHBV-PTX-NPs aqueous solution was freeze-dried using Freeze Dry System (Labconco, Kansas, MO, USA) to ensure the stability of the physical properties of the final NPs. NPs can be readily marked by adding fluorescent dyes (such as DIR and FITC) to the organic phase before emulsification.

### PDA coating

6 mg of the PHBV-PTX-NPs (prepared at "Preparation of PHBV-PTX-NPs") were re-suspended in 6 mL of Tris buffer (10 mM, pH 8.5). 3 mg of dopamine was added to the suspension and stirred continuously at room temperature. The color of the solution changed to dark gray after stirring for 3 h, proving that dopamine was polymerized successfully. The pH value of the whole system was adjusted to neutral by adding hydrochloric acid aqueous solution (0.1 M, pH 1) in order to terminate the polymerization of dopamine. Next, the suspension was centrifuged at 10,000 rpm for 10 min to obtain PDA-PHBV-PTX-NPs.

### Conjugation of RGD with PDA-PHBV-PTX-NPs

The RGD was used as a functional targeting ligand to bind to the PDA coating through Michael addition [28]. In short, the PDA-PHBV-PTX-NPs (prepared at "PDA coating") were re-suspended in Tris buffer (10 mM, pH 8.5) containing 2 mg/mL of RGD ligands. It was continuously stirred and incubated at room temperature for 0.5 h. The free RGD in the supernatant was then eliminated post-centrifugation at 10,000 rpm for 15 min (Sigma 3K30 Centrifuge, Germany). The sample was freeze dried at − 60 °C for 48 h to acquire RGD-PDA-PHBV-PTX-NPs lyophilized powder. The content of RGD on the NPs was determined based on Bradford protein method [29].

### Evaluation of size and morphology of NPs

Particle size, Zeta potential and polydispersion index (PDI) of PHBV-PTX-NPs, PDA-PHBV-PTX-NPs and RGD-PDA-PHBV-PTX-NPs were determined by dynamic light scattering- autosizer (DLS, Zeta Plus Brookaven Instruments, Holtsville, NY, USA) method. The morphology of the samples were investigated by scanning electron microscope (SEM). The samples were adhered to the gold conductive adhesive (JEOL, Ltd., Tokyo, Japan), and was tested by the SEM (ZEIS, Ltd., Germany). In addition, the morphology of the samples were studied by transmission electron microscope (TEM). A drop of samples were dropped on the copper grid, air-dried and then was detected under TEM (EOL Ltd., Tokyo, Japan).

### Entrapment efficiency and drug loading efficiency

The entrapment efficiency (EE) and drug loading efficiency (LE) of RGD-PDA-PHBV-PTX-NPs were determined by HPLC (LC 1200, Agilent Technologies, Santa Clara, CA, USA) methods [30]. In brief, 10 mg of RGD-PDA-PHBV-PTX-NPs were dissolved in 2 mL of methanol under vigorous vortexing. The dissolved PTX in methanol was determined by HPLC using a Diamonsil C_18_ column (250 mm × 4.6 mm, 5 μm; Agilent Technologies, CA, USA). The mobile phase was composed of methanol, acetonitrile and methanol at a volume ratio of 525:225:250 (v/v). The flow rate was 1.0 mL/min, and the UV detection wavelength was 227 nm. The relative concentration of PTX in the suspension was calculated from the standard curve *y* = 18,158,852.68 × *x* − 68,768.24 and *R*^2^ = 0.9995 (where *x* is the PTX concentration [mg/mL] and *y* is the peak area). The LE and EE of RGD-PDA-PHBV-PTX-NPs were calculated by the following equations, respectively.

$$ {\text{LE}}\%  = \frac{{{\text{Weight}}\;{\text{of}}\;{\text{PTX}}\;{\text{in}}\;{\text{NPs}}}}{{{\text{Weight}}\;{\text{of}}\;{\text{NPs}}}} \times 100\% $$

$$ {\text{EE}}\%  = \frac{{{\text{Weight}}\;{\text{of}}\;{\text{PTX}}\;{\text{in}}\;{\text{NPs}}}}{{{\text{Weight}}\;{\text{of}}\;{\text{the}}\;{\text{feeding}}\;{\text{PTX}}}} \times 100\%  $$

### Fourier transform infrared spectroscopy

Solid properties of the samples were determined by fourier transform infrared spectrometer (FTIR) (Nicolet, Madison, WI, USA). The wavenumber range 4000–400/cm was detected with resolution 1/cm.

### X-ray diffraction

Physical properties of the samples were determined by X–ray diffractometer (XRD) (Philips, Amsterdam, The Netherlands), The diffraction patterns of the samples were obtained in the range of 5–80° by X–ray diffraction.

### Differential scanning calorimetry

Thermodynamic changes related to the morphological of the samples were detected by differential scanning calorimetry (DSC) (Shimadzu, Kyoto, Japan). The samples were heated from 50 °C to 400 °C at a 10 °C/min of constant heating rate in nitrogen.

### Thermogravimetry

The thermal stability of the samples were checked by thermogravimetry (TG) analyzer (Diamond TG/DTA PerkinElmer, Waltham, MA, USA). The samples were heated from 50 °C to 400 °C at a 10 °C/min of constant heating rate in nitrogen.

### Stability evaluation

Physical stability of the PHBV-PTX-NPs, PDA-PHBV-PTX-NPs and RGD-PDA-PHBV-PTX-NPs in aqueous PBS solution was evaluated by placing them at 37 °C for 14 days. During this period, the particle size distribution of NPs in PBS was measured at a certain time interval.

### Hemolysis experiment

Hemolysis test was used to investigate the irritation and biocompatibility of nano-drugs to erythrocytes. 2 mL of fresh blood was taken from the fundus plexus vein of Kunming (KM) mice. Erythrocytes were collected by centrifugation at 3000 rpm and 4 °C for 15 min. The isolated red blood cells were prepared into 4% normal saline suspension. 500 μL of saline isotonic solution of PHBV-PTX-NPs, PDA-PHBV-PTX-NPs and RGD-PDA-PHBV-PTX-NPs (PTX concentrations of 1, 0.5, 0.25, 0.125, 0.0625 mg/mL, respectively), were mixed with 500 μL of 4% erythrocyte suspension, and then incubated at 37 °C for 4 h. The supernatant was collected by centrifugation at 3000 rpm and 4 °C for 10 min. The absorbance value was measured at 540 nm by a microplate reader (Molecular Devices, LLC., Sunnyvale, CA, USA). In addition, the erythrocyte suspension treated with normal saline was used as the negative control, and the erythrocyte suspension treated with Triton X-100 was used as the positive control. The percentage of hemolysis was calculated by the following formula:

$$  {\text{Hemolysis\, rate}}\% = \frac{{{\text{Sample\,absorbance}} - \;{\text{Negative\,control}}}}{{{\text{Positive\,control}}\; - \;{\text{Negative\,control}}}} \times 100\% $$

### In vitro* PTX release*

In vitro release of the pure PTX, PHBV-PTX-NPs, PDA-PHBV-PTX-NPs and RGD-PDA-PHBV-PTX-NPs (3 mL, 0.3 mg of PTX) in phosphate buffer saline (PBS) under different pH conditions was analyzed by dialysis bag diffusion method. In brief, the samples were placed in a regenerated cellulose dialysis bag (MWCO, 8000–14,000 Da). Then, the dialysis bag sealed by the clips at both ends was placed in a beaker containing 200 mL of release medium PBS (pH = 5, 6.5 and 7.4). The whole system was incubated at 37 °C by stirring at 150 rpm. At a specific time point, 3 mL of dialysis medium was taken out for HPLC analysis. Meanwhile, the same volume of fresh medium was supplemented. Each samples were analyzed in triplicates. The amount of released PTX was analyzed with HPLC at 227 nm.

### Cell culture and cellular viability evaluation

The cells were cultured in DMEM (HepG2) and RPMI-1640 medium (SMMC-7721 and L02), containing 10% fetal bovine serum and 1% penicillin streptomycin and maintained at 37 °C and 5% CO_2_ moist atmosphere. In vitro cytotoxicity experiments were conducted by the MTT assay. In brief, HepG2, SMMC-7721 and L02 cells were inoculated at a density of 8000 cells per well and cultured in 96-well plates for 24 h. To evaluate the cytotoxicity of RGD-PDA-PHBV-NPs (PTX-free), the effect on the viability of L02 cells was determined. Next, the medium containing different concentrations of free PTX, PHBV–PTX-NPs, PDA–PHBV–PTX-NPs, RGD–PDA–PHBV–PTX-NPs and RGD-PDA–PHBV-NPs were used instead of the previous medium and incubated at 37 °C for 48 h in the dark. 10 μL of MTT (5 mg/mL) solution was added to each pore and incubated for 4 h. The medium was replaced with 150 μL of DMSO, and then was incubated for 10 min. A microplate reader (Molecular Devices, LLC., Sunnyvale, CA, USA) was used to measure the absorbance at 490 nm. The cell inhibition rate is calculated as follows:

$$   {\text{Cell\,inhibitory\,rate}}\%\; = \;\left( {1 - \frac{{A_{S} }}{{A_{c} }}} \right) \times 100\% $$

where *A*_*S*_ is the average absorbance value of the experimental group, and *Ac i*s the average absorbance value of the control group.

### Cell uptake

Cellular uptake behavior of HepG2 and SMMC-7721 cells was detected by a confocal laser scanning microscope (CLSM) (Zeiss, Ulm, Germany). In brief, the cells were inoculated in a 12-well plate with glass slides at a density of 1 × 10^5^ per well and then incubated at 37 °C and 5%CO_2_ for 24 h. The medium was sucked out and replaced with fresh DMEM medium including different samples (PHBV-PTX-NPs, PDA-PHBV-PTX-NPs and RGD-PDA-PHBV-PTX-NPs). Next, the cells were washed with PBS, fixed with 4% paraformaldehyde (PFA) for 10 min, and followed by DAPI staining and PBS washes. The images of drug distribution were collected immediately through the CLSM.

### Xenograft tumor model

All animal experiments were implemented in accordance with the Guidelines for the Care of Northeast Forestry University. Permission for animal experiments was obtained from the Ethics Committee of the Northeast Forestry University. BALB/c male nude mice (5–6 weeks, 18–20 g, for in vivo distribution and therapeutic treatment) and KM male mice (5–6 weeks, 20–22 g for hemolysis experiment) were purchased from Beijing Vital River Laboratory Animal Technology Co., Ltd., (Beijing, China). The tumor model based on BALB/c nude mice was established by subcutaneous injection of 5 × 10^6^ HepG2 cells (0.2 mL cell suspension) into the right back of BALB/c nude mice. The mice were administrated when the tumor size reached approximately 100 mm^3^. The equation for calculating tumor volume (mm^3^) was as follows: volume = *ab*^2^/2, where* a* and *b* represent the width and length of tumor, respectively.

### In vivo* biodistribution study*

The DIR, PTX and PHBV were added to the organic phase of dichloromethane at a 1:40:120 ratio and were fully dissolved by ultrasound. DIR-labelled PHBV-PTX-NPs were prepared by emulsion solvent evaporation method. The method is consistent with that described in "Preparation of PHBV-PTX-NPs". The free DIR dye was then separated by ultracentrifugation and verified by the detection of fluorescence in the supernatant. The tumor-bearing mice were randomly divided into 4 groups. Free DIR, PHBV-PTX/DIRNPs, PDA-PHBV-PTX/DIRNPs and RGD-PDA-PHBV-PTX/DIRNPs were injected into the caudal vein at a dose of 2 mg/kg PTX (PTX: DIR = 40: 1). Fluorescence images of the whole body were recorded at 1, 4, 8, 12, 24 and 48 h using an IVIS Lumina III (PerkinElmer, Waltham, MA, USA) under isoflurane anesthesia. After 48 h, all the mice were sacrificed to collect organs (heart, liver, spleen, lung and kidney) and tumors for in vitro imaging and quantitative analysis. Quantitative analysis was carried out by using a Living Image^®^ 4.2 software (Caliper Life Sciences, Hopkinton, MA, USA). All the mice were imaged at 780 nm excitation wavelengths using the same instrument.

### In vivo* antitumor efficacy*

The HepG2 tumor-bearing BALB/c nude mice were randomly divided into 3 groups with 5 mice per group: a negative group treated with physiological saline; a positive group treated with PTX (4 mg/kg of body weight, thrice per week) and an RGD-PDA-PHBV-PTX-NPs group (14.21% (W/W) PTX loading, 28 mg/kg of body weight, thrice per week). All the mice were administrated through the tail vein for 14 days in total. Mice body weight and tumor volume were measured daily throughout the treatment. At the end of treatment, all the mice were sacrificed to collect main organs. Next, these organs, including hearts, livers, spleens, lungs and kidneys were fixed with 4% PFA solution and embedded in paraffin for H&E staining.

### Statistical analysis

The results of statistical analysis were obtained by using the GraphPad Prism 5.0 software (GraphPad, SanDiego, CA, USA). The Student's t-test and one-way ANOVA were employed to assess the statistical significance. A *p*-value < 0.05 was considered significant. *p* < 0.05 (*), *p* < 0.01 (**), and *p* < 0.001 (***).

## Results and discussion

### Optimization of preparation process of nanoparticles

**Fig. 1:**
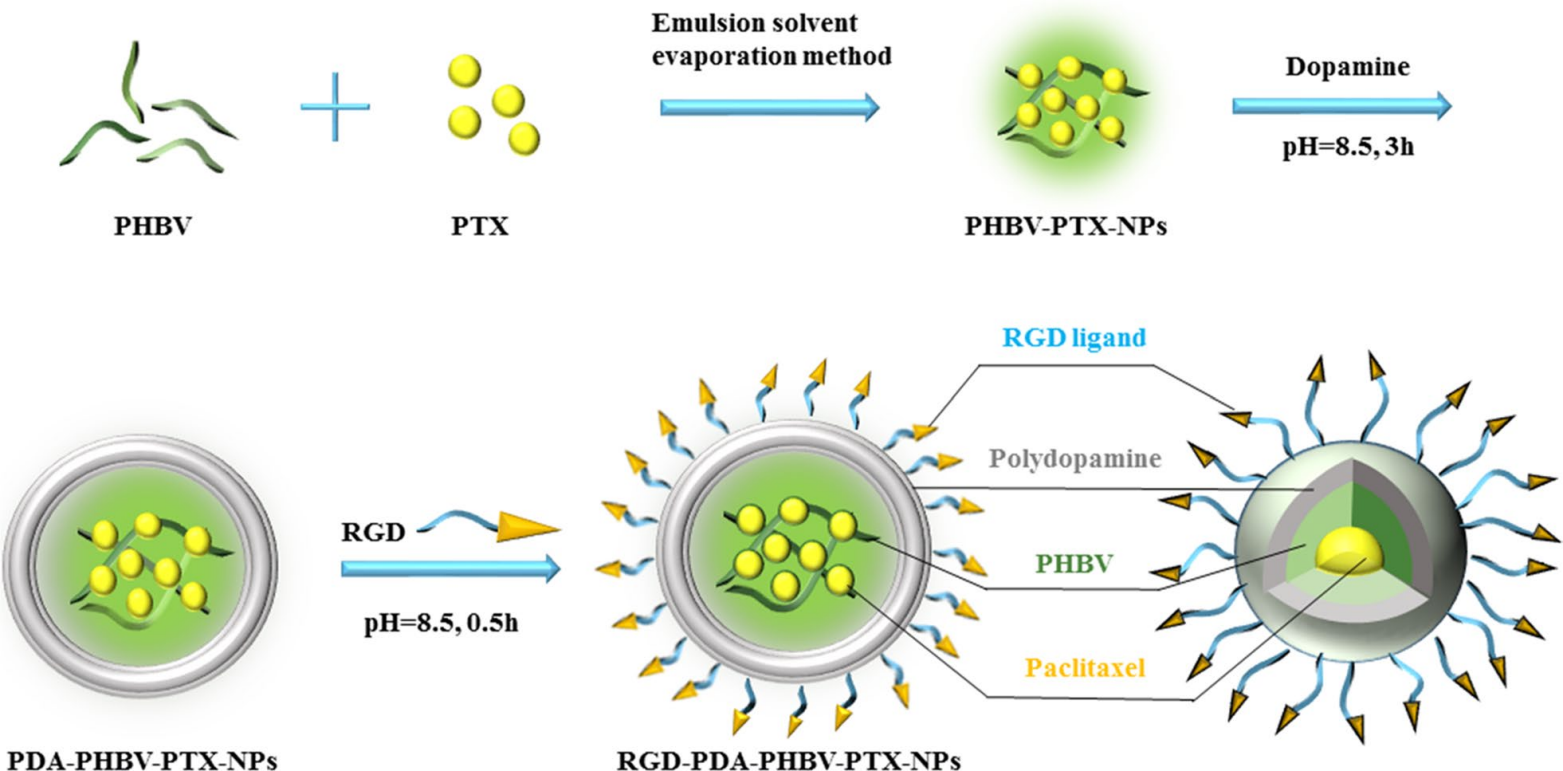
Schematic diagram of the preparation process of RGD-PDA-PHBV-PTX-NPs. PHBV, ploy (3-hydroxybutyrate-co-3-hydroxyvalerate)

In this work, we firstly prepared PHBV-PTX-NPs by emulsifying solvent evaporation. Next, PHBV-PTX-NPs were re-suspended in dopamine solution to form a compact film on the surface. In alkaline aqueous solution, dopamine can be oxidized and polymerized to form a dense shell. When the color of the suspension turns dark, it represents that the dopamine has successfully polymerized. Finally, RGD-PDA-PHBV-PTX-NPs were obtained by the Michael addition between RGD and PDA coating. The preparation process of RGD-PDA-PHBV-PTX-NPs is exhibited in Fig. 1.

**Fig. 2:**
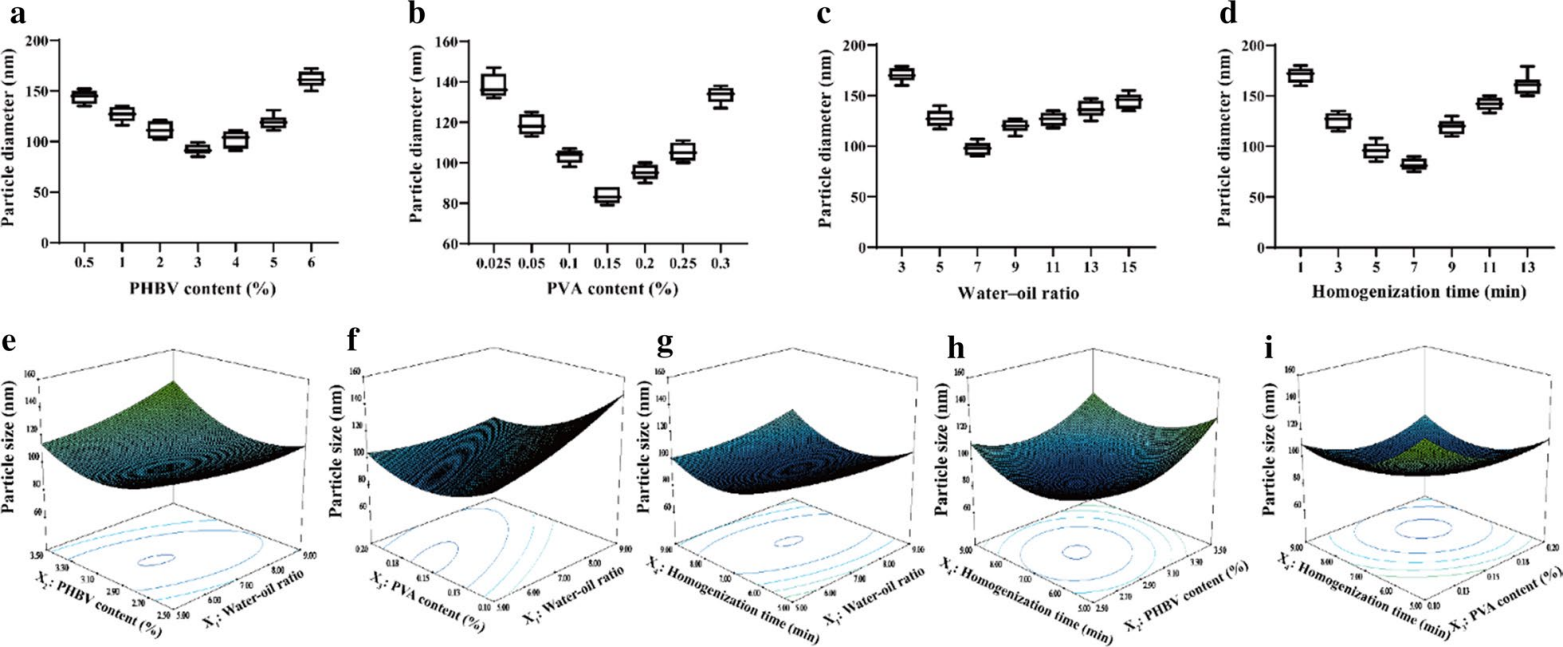
Effects of PHBV content (**a**), PVA content (**b**), water–oil ratio (**c**) and homogenization time (**d**) on the preparation of PHBV-PTX-NPs by single factor design (n = 5). Three-dimensional response surface diagram of the interaction between water–oil ratio and PHBV content (**e**), the interaction between water–oil ratio and PVA content (**f**), the interaction between water–oil ratio and homogenization time (**g**), the interaction between PHBV content and homogenization time (**h**), the interaction between PVA content and homogenization time (**i**). PHBV, ploy (3-hydroxybutyrate-co-3-hydroxyvalerate); PVA, polyvinyl alcohol

In the first set of experiments, the single factor and response surface methods were used to determine the best preparation conditions of PHBV-PTX-NPs. In the processing of PHBV-PTX emulsion, we found that PHBV content (%), PVA content (%), water–oil ratio, (v/v), homogenization time (min), homogenization pressure (MPa) and homogenization frequencies (tines) at certain pressure levels have significant effects on particle size. In the first stage of high-speed homogenization test, four factors including PHBV content (%), PVA content (%), water–oil ratio, (v/v), homogenization time (min) were selected for single factor experiments. Taking the particle size as the screening standard, the best preparation process of PHBV-PTX-NPs was determined. The optimum conditions obtained by single factor optimization are as follows: PHBV content 3%, PVA content 0.15%, water–oil ratio 7:1 and homogenization time 7 min (Fig. 2a–d).

According to the results of single-factor experiments, the preparation conditions of PHBV-PTX-NPs were further optimized by response surface method. Design Expert 8.0.6 software (Stat-Ease, Inc., Minneapolis, MN, USA) was used to optimize the experimental design. The experiment of 4 factors and 5 levels was carried out according to the principle of Central Composite Design (CCD). Taking the particle size of PHBV-PTX-NPs as the evaluation index, the factors such as water–oil ratio (*X*_*1*_), PHBV content (*X*_*2*_), PVA content (*X*_*3*_) and homogenization time (*X*_*4*_) were selected at the best level. Table 1 shows the coded and actual values of the variables. On the basis of the CCD matrix listed by Design-Expert software, 30 experiments were carried out to estimate the sum of squares of pure errors (Table 2). The response variables and test variables were related by the second-order polynomial equation for the PHBV-PTX-NPs size as follows:

$$\begin{aligned}   Y = &\,  80.17 + 9.54X_{1}  + 1.54X_{2}  - 18.54X_{3} - 7.67X_{4}  + 7.23X_{1} X_{2}  - 9.52X_{1} \;X_{3}  \hfill \\    &+ 5.19X_{1} X_{4}  + 0.53X_{2} X_{3}  - 2.69X_{2} X_{4}  - 6.31X_{3} X_{4}  + 4.74X_{1}^{2}  + 15.61X_{2}^{2}  + 13.99X_{3}^{2}  + 16.46X_{4}^{2}  \hfill \\  \end{aligned}  $$

**Table 1:** The coded and actual values of the variables used in central composite design

Symbol	Independent variables	Levels				
		− α	− 1	0	1	+ α
A	Water–oil ratio (v/v)	3:1	5:1	7:1	9:1	11:1
B	Polymer content (%)	2	2.5	3	3.5	4
C	PVA content (%)	0.05	0.1	0.15	0.2	0.25
D	Homogenization time (min)	3	5	7	9	11
Constraints	Dependent variables (Units)					
Minimize	Y = particle size (nm)					

*PVA* polyvinyl alcohol

**Table 2:** Statistical ANOVA analysis of Quadratic model

Source	Sum of square	Degree of freedom	Mean square	*F*-value	*p*-value ^b^	
Model^a^	30,567.71	13	2351.36	302.94	< 0.0001	
*X*_*1*_	591.56	1	591.56	76.22	< 0.0001	
*X*_*2*_	5962.07	1	5962.07	768.14	< 0.0001	
*X*_*3*_	2884.03	1	2884.03	371.57	< 0.0001	
*X*_*4*_	3828.79	1	3828.79	493.29	< 0.0001	
*X*_*1*_* X*_*2*_	835.21	1	835.21	107.61	< 0.0001	
*X*_*1*_* X*_*3*_	1451.61	1	1451.61	187.02	< 0.0001	
*X*_*1*_* X*_*4*_	430.56	1	430.56	55.47	< 0.0001	
*X*_*2*_* X*_*4*_	115.56	1	115.56	14.89	0.0014	
*X*_*3*_* X*_*4*_	637.56	1	637.56	82.14	< 0.0001	
*X*_*1*_^2^	615.60	1	615.60	79.31	< 0.0001	
*X*_*2*_^2^	6685.72	1	6685.72	861.37	< 0.0001	
*X*_*3*_^2^	5366.40	1	5366.40	691.39	< 0.0001	
*X*_*4*_^2^	7433.52	1	7433.52	957.72	< 0.0001	

Credibility analysis of the regression equations						
Standard deviation	Mean	Coefficient of variation	*R*^*2*^	Adjust* R*^*2*^	Predicted* R*^*2*^	Adequacy precision
2.79	120.81	2.31	0.996	0.9927	0.9819	57.161

The results were obtained with Design Expert 8.0 software

^a^ *X*_*1*_: water–oil ratio; *X*_*2*_: PHBV content (%); *X*_*3*_: PVA content (%); *X*_*4*_: homogenization time (min); *Y*: particle size (nm)

^b^ *p*-value < 0.05 consider as statistically significant

The “Lack of fit *p* value” of 0.1565 > 0.05 indicates that the lack of fit is not significantly related to the pure error, which shows that the quadratic regression model fits well with actual situation. The *R*^2^ value of the equation is 0.996, and the correlation coefficient is close to 1, indicating that the prediction result of the model is relatively accurate. The correction determination coefficient *R*_*adj*_^2^ is 0.9927, indicating that there is a good linear correlation among independent variables. Response surface plots in contour and three-dimensional plots were used to identify the relationship between factors and responses (Fig. 2e–i). The optimum conditions for the point prediction by the response surface software were: 6.7:1 water–oil ratio, 2.99% PHBV content, 0.16% PVA content and 7.45 min homogenization time. Under the conditions of the point prediction, the particle size of PHBV-PTX-NPs was 77.3 nm. In the second stage of high-pressure homogenization test, two factors including homogenization pressure and homogenization times were selected for single factor experiment. The optimal conditions are as follows: homogenization pressure 80 MPa and homogenization times 7 times. Finally, the particle size of PHBV-PTX-NPs obtained under the optimum conditions was 66.7 ± 5.2 nm.

### Particle morphology, size and Zeta potential

**Fig. 3:**
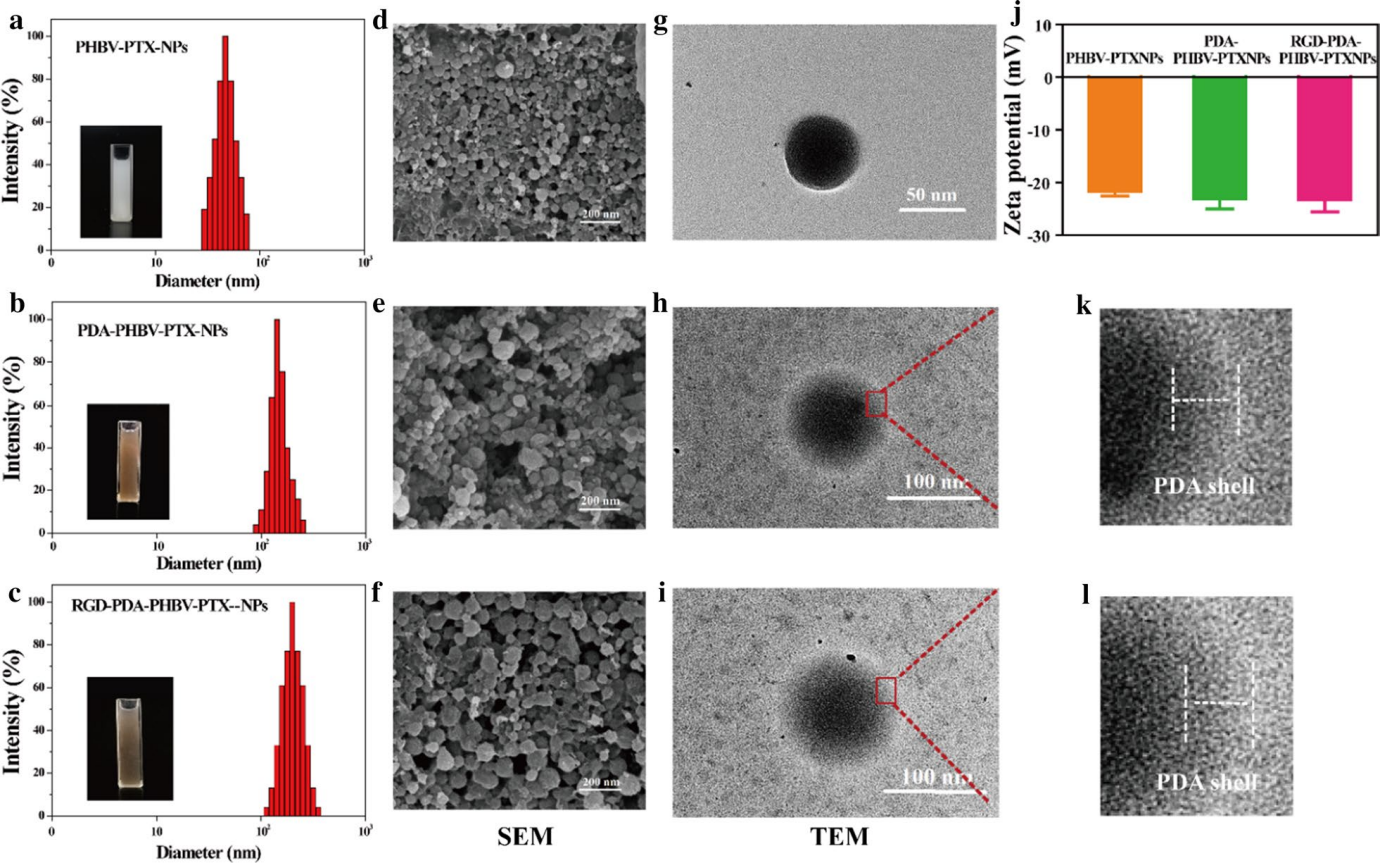
DLS size distribution histograms of (**a**) PHBV-PTX-NPs, (**b**) PDA-PHBV-PTX-NPs and (**c**) RGD-PDA-PHBV-PTX-NPs. The SEM images of (**d**) PHBV-PTX-NPs, (**e**) PDA-PHBV-PTX-NPs and (**f**) RGD-PDA-PHBV-PTX-NPs. The TEM images of (**g**) PHBV-PTX-NPs, (**h**, **k**) PDA-PHBV-PTX-NPs and (**i**, **l**) RGD-PDA-PHBV-PTX-NPs. (I) Zeta potential of samples data are expressed as means ± SD (n = 3). PHBV, ploy (3-hydroxybutyrate-co-3-hydroxyvalerate); PTX, paclitaxel; PDA, polydopamine; NPs, nanoparticles

The tumor accumulation of NPs mainly relies on the enhanced permeability and retention (EPR) effect. In the EPR-mediated tumor targeting, drug delivery systems based on polymer conjugates, micelles and liposomes, are typically have sizes ranging from 5 to 200 nm [15]. We thus selected particle size as the evaluation index in this study. Figure 3a–c showed that the hydrodynamic diameters of PHBV-PTX-NPs, PDA-PHBV-PTX-NPs and RGD-PDA-PHBV-PTX-NPs were 66.7 ± 5.2 nm, 113.5 ± 7.4 nm and 124.6 ± 7.7 nm, respectively. Furthermore, they had small PDI values. The SEM images of PHBV-PTX-NPs, PDA-PHBV-PTX-NPs and RGD-PDA-PHBV-PTX-NPs were showed in Fig. 3d–f. The PHBV-PTX-NPs obtained under the optimum conditions exhibited a uniform spherical morphology with a particle size of ~ 70 nm (Fig. 3d). The PDA-PHBV-PTX-NPs and RGD-PDA-PHBV-PTX-NPs were approximately spherical shape, and the particle sizes of these two NPs were slightly larger than PHBV-PTX-NPs.

The morphology of NPs was showed in Fig. 3g–i. All of the PHBV-PTX-NPs, PDA-PHBV-PTX-NPs and RGD-PDA-PHBV-PTX-NPs were spherical. The shell with a certain thickness could be observed apparently on the surface of PDA-PHBV-PTX-NPs and RGD-PDA-PHBV-PTX-NPs, suggesting PDA was successfully deposited on the surface of PHBV-PTX-NPs by oxidative polymerization (Fig. 3k, l). As shown in Table 3, the sizes of PDA-PHBV-PTX-NPs and RGD-PDA-PHBV-PTX-NPs increased by nearly 50 nm compared to that of PHBV-PTX-NPs, which may be attributed to the thin PDA film formed around the PHBV-PTX-NPs.

**Table 3:** Characterization of PHBV-PTX-NPs, PDA-PHBV-PTX-NPs and RGD-PDA-PHBV-PTX-NPs

Samples (n = 3)	Size (nm)	PDI	LE (%)	EE (%)
PHBV-PTX-NPs	66.7 ± 5.2	0.142	21.48 ± 0.83	74.42 ± 1.21
PDA-PHBV-PTX-NPs	113.5 ± 7.4	0.153	15.33 ± 0.74	73.45 ± 1.03
RGD-PDA-PHBV-PTX-NPs	124.6 ± 7.7	0.163	14.21 ± 0.66	71.21 ± 1.06

Data presents as mean ± standard deviation

*PDI* polydispersion index, *LE* drug loading efficiency, *EE* encapsulation efficiency

The Zeta potentials of PHBV-PTX-NPs, PDA-PHBV-PTX-NPs and RGD-PDA-PHBV-PTX-NPs are − 21.7 ± 3.7 mV, − 23.1 ± 4.2 mV and − 23.5 ± 4.5 mV, respectively (Fig. 3j). The negative charge on the surface of NPs has better stability in circulatory system in vivo [31, 32]. The LE of PHBV-PTX-NPs, PDA-PHBV-PTX-NPs and RGD-PDA-PHBV-PTX-NPs were 21.48% ± 0.83%, 15.33% ± 0.74% and 14.21% ± 0.66%, respectively. The EE of RGD-PDA-PHBV-PTX-NPs (71.21% ± 1.06%) was slightly lower than that of PHBV-PTX-NPs (74.42% ± 1.21%) and PDA-PHBV-PTX-NPs (73.45% ± 1.03%), which caused by the loss of loaded drugs when ligand RGD was coupled with NPs.

### Solid-state study

**Fig. 4:**
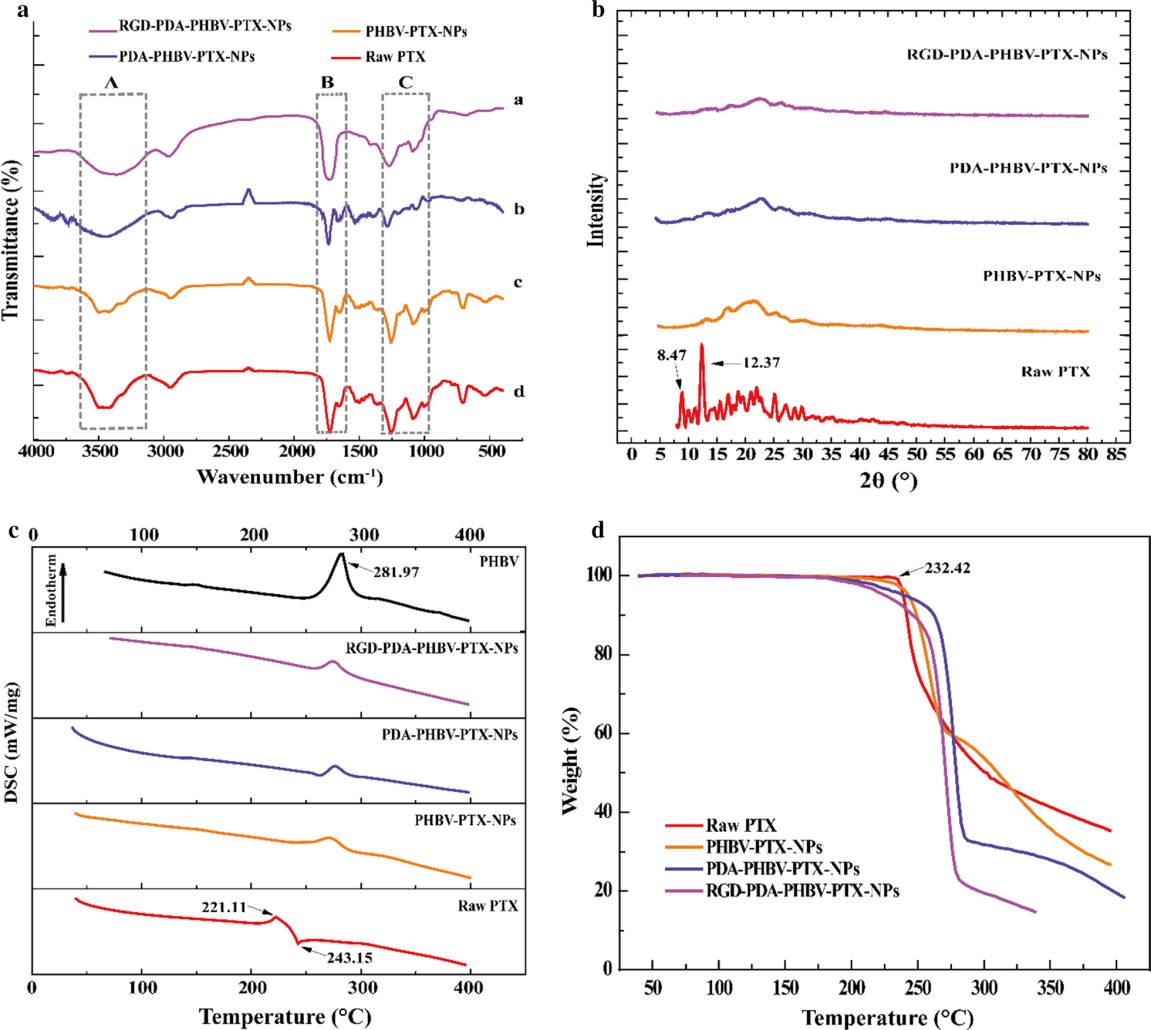
**a** Fourier transform infrared spectra. **b** X-ray diffraction curves of samples. **c** Differential scanning calorimetric curves of samples. **d** Thermo gravimetric curves of samples. PHBV, ploy (3-hydroxybutyrate-co-3-hydroxyvalerate); PTX, paclitaxel; PDA, polydopamine; NPs, nanoparticles

The composition of surface chemical groups of NPs was verified by FTIR analysis. The FTIR spectra of raw PTX, PHBV-PTX-NPs, PDA-PHBV-PTX-NPs and RGD-PDA-PHBV-PTX-NPs were showed in Fig. 4a. Dopamine modified NPs (PDA-PHBV-PTX-NPs and RGD-PDA-PHBV-PTX-NPs) exhibited a strong absorption peak at 3430 cm^−1^, which could be attributed to the stretching vibration of hydroxyl groups including water adsorbed on the surface. In addition, the 3430 cm^−1^ bands of PDA-PHBV-PTX-NPs and RGD-PDA-PHBV-PTX-NPs were significantly higher than that of PHBV-PTX-NPs, which may owe to the O–H and N–H stretching modes of PDA and RGD. In the infrared spectrum of PHBV-PTX-NPs, the 1728 cm^−1^ peak was designated as the carbonyl band of PHBV. However, the 1728 cm^−1^ peak of PDA-PHBV-PTX-NPs decreased significantly, indicating that the PDA is coated on PHBV-PTX-NPs. Additionally, the absorption band of RGD-PDA-PHBV-PTX-NPs at 1728 cm^−1^ was increased, which is consistent with the existence of carbonyl groups in RGD, indicating that the targeted groups are coupled on PDA-PHBV-PTX-NPs. The binding amount of RGD on the surface of NPs was determined to be 56.32 μg/mg by the Bradford method.

The crystallinity of raw PTX, PHBV-PTX-NPs, PDA-PHBV-PTX-NPs and RGD-PDA-PHBV-PTX-NPs were studied by XRD. As shown in Fig. 4b, the X-ray diffraction pattern of PTX had two spikes at 2*θ* diffraction angles of 8.74°and 12.37°, respectively, indicating that PTX formed a crystal structure. Compared with raw PTX, the crystallinity of PHBV-PTX NPs encapsulated by PHBV significantly decreased. In the XRD curve of RGD-PDA-PHBV-PTX-NPs, there is no diffraction characteristic peak of crystalline PTX, indicating that PTX exists in an amorphous form in the particle matrix. The peaks shown in the XRD curves of PHBV-PTX-NPs, PDA-PHBV-PTX-NPs and RGD-PDA-PHBV-PTX-NPs were not caused by the PTX, but by the polymerization of polymers.

Figure 4c showed a DSC thermogram of the samples. The DSC map was obtained to determine whether PTX was amorphous dispersed in the polymer matrix, and to detect the structural changes of the polymers. The DSC curve of PTX had the peak at 221.11 °C and 243.15 °C, which was expressed as the melting point and decomposition temperature, respectively. The DSC pattern of polymers PHBV showed that the melting point was 281.97 °C. The melting point peak of PTX was not appeared in the DSC curves of PHBV-PTX-NPs, PDA-PHBV-PTX-NPs and RGD-PDA-PHBV-PTX-NPs, indicating that PTX exists in the particles in an amorphous form, which is consistent with the XRD results. The change of melting point in the DSC curve of the samples was caused by the degree of polymerization of NPs rather than PTX. The advantage of this amorphous form drug dispersed in the polymer matrix is that it increases the solubility in water, thereby increasing the bioavailability of poorly water-soluble drug.

The TG analysis was used to determine the relationship between sample quality and temperature. The TG curve of the weight change of the samples caused by water loss was shown in Fig. 4d. The TG curve of raw PTX showed that weightlessness occurred when the temperature was rose to 232.42 °C, and the total weight loss rate was 65%. The weight of RGD-PDA-PHBV-PTX-NPs began to lose at 182 °C, and the total weight loss rate was 86%. This result demonstrated that RGD-PDA-PHBV-PTX-NPs had the smaller particle size than raw PTX. The small particle size means high specific surface area and strong specific surface energy, which generally lead to the easier evaporation and earlier decomposition energy.

### Stability and safety evaluation

**Fig. 5:**
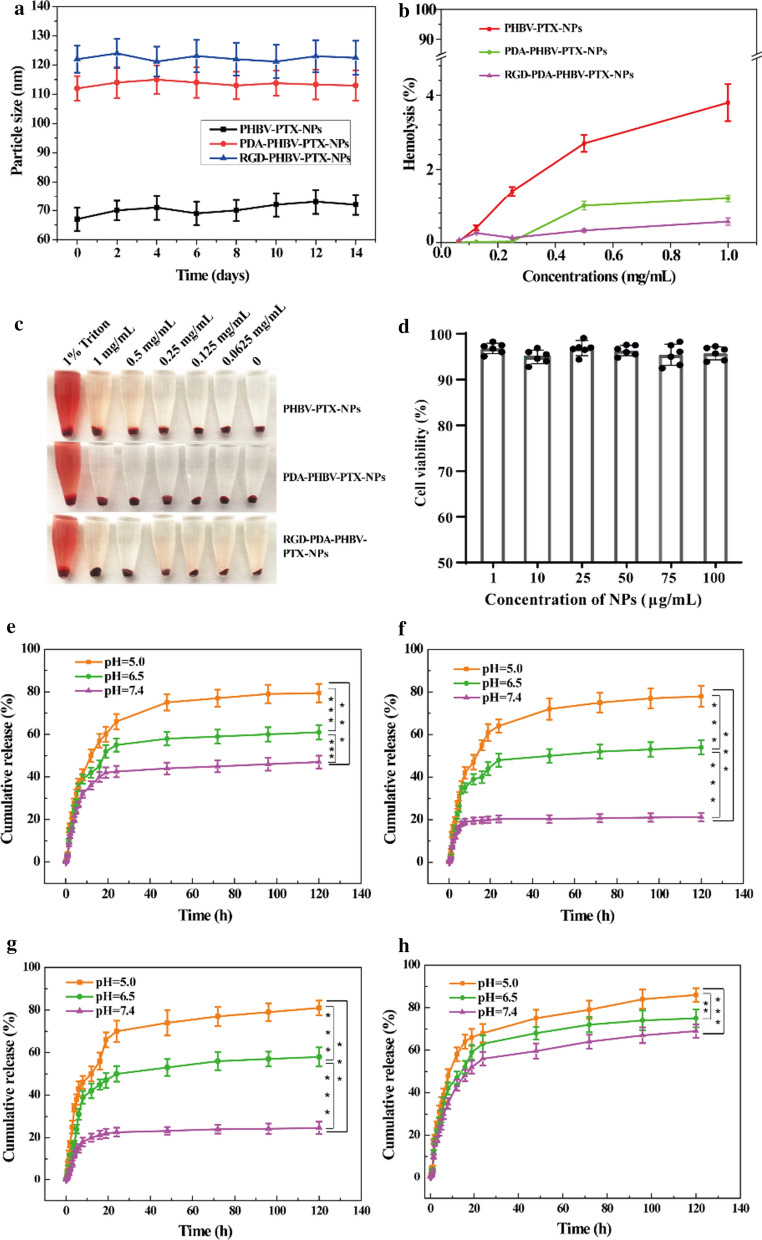
**a** The stability of nanoparticles in PBS solution (mean ± SD, n = 3). **b** Hemolysis rate of different nanoparticle*s *in vitro (mean ± SD, n = 3). **c** Effects of different nanoparticles on hemolysis in vitro*.* **d** Cytotoxicity against L02 cells after incubation with different concentrations of RGD-PDA-PHBV-NPs (PTX-free) for 48 h. In vitro drug release profile of PHBV-PTX-NPs (**e**), PDA-PHBV-PTX-NPs (**f**), RGD-PDA-PHBV-PTX-NPs (**g**) and raw PTX (**h**) in PBS with different pH value (n = 3). One way ANOVA, *p < 0.05; **p < 0.01, ***p < 0.001. PHBV, ploy (3-hydroxybutyrate-co-3-hydroxyvalerate); PTX, paclitaxel; PDA, polydopamine; NPs, nanoparticles

The stability of RGD-PDA-PHBV-PTX-NPs in physiological condition is pivotal for further application in vivo. The stability of particle size of PHBV-PTX-NPs, PDA-PHBV-PTX-NPs and RGD-PDA-PHBV-PTX-NPs in PBS solution was monitored within 14 days. Figure 5a showed that RGD-PDA-PHBV-PTX-NPs had a favorable redispersion stability. The average size of NPs remained almost stable in the 14-day experiment. To estimate the biocompatibility of the NPs, red blood cells were used to detect their hemolytic activity [33]. The results showed that all the tested NPs showed moderate hemolytic activity (Fig. 5b, c). Furthermore, the cytotoxicity of the RGD-PDA-PHBV-NPs (PTX-free), as a drug-delivery vehicle, was evaluated by MTT assay by using normal human L02 hepatocytes. No obvious cytotoxicity was observed post the RGD-PDA-PHBV-NPs treatment with the increasing PHBV concentrations for 48 h (Fig. 5d). Although the concentration of RGD-PDA-PHBV-NPs was increased to 100 μg/mL, the cell viability still kept at 96.8%. These results demonstrated that the RGD-PDA-PHBV-NPs prepared with PHBV has the favorable biocompatibility.

### pH-sensitive drug release in vitro

To explain the differences in the release process of different nanoparticles, the PTX release test in vitro was carried out. The cumulative dissolution curve of the NPs was showed in Fig. 5e–h. At pH 7.4, the PDA-PHBV-PTX-NPs and RGD-PDA-PHBV-PTX-NPs released 21.2% and 24.7% of PTX within 120 h, respectively (Fig. 5f, g). Zhu et al. [34], in 2016, developed that the docetaxel (DTX)-loaded formulations using polydopamine-modified NPs synthesized from D-α-tocopherol polyethylene glycol 1000 succinate-poly(lactide) (pD-TPGS-PLA/NPs). After 120 h, the cumulative release rate of pD-TPGS-PLA NPs loaded with DTX reached up to 43%, which is apparently higher than that of RGD-PDA-PHBV-PTX-NPs. At pH 6.5, the release accumulation of PDA-PHBV-PTX-NPs and RGD-PDA-PHBV-PTX-NPs increased to 54.1% and 58.2%, respectively. As expected, in the environment of pH 5.0, the release accumulation of PDA-PHBV-PTX-NPs and RGD-PDA-PHBV-PTX-NPs had the further increase (78.3% and 81.5%, respectively). There was a little burst release of PHBV-PTX-NPs within the first 8 h. However, the initial release amount of PHBV-PTX-NPs decreased post-modification with PDA (Fig. 5e). Moreover, the release curve of PTX was showed in Fig. 5h and the release trend was almost invariable within the first 10 h.

The results suggested that the PDA-coated NPs showed higher PTX release rate at pH 5.0–6.5 than physiological pH 7.4 in vivo. Because the deposited PDA films are able to retain the structure of NPs under physiological pH 7.4, but unlock the channels under acidic conditions. The RGD-PDA-PHBV-PTX-NPs can control drug release through the change of pH value in vivo, thereby minimizing the leakage of PTX. The range of pH values in inclusion bodies and lysosomes of tumor cells is 5.0–6.5, and that of tumor microenvironment is 6.5–7.2 [35]. PH-sensitive NPs can retain drug during humoral circulation and actively release drug around the tumor site or in lysosomes of tumor cells [36]. Therefore, we believe that the RGD-PDA-PHBV-PTX-NPs can remarkably reduce the potential damage to normal cells.

### In vitro cellular cytotoxicity and uptake

**Fig. 6:**
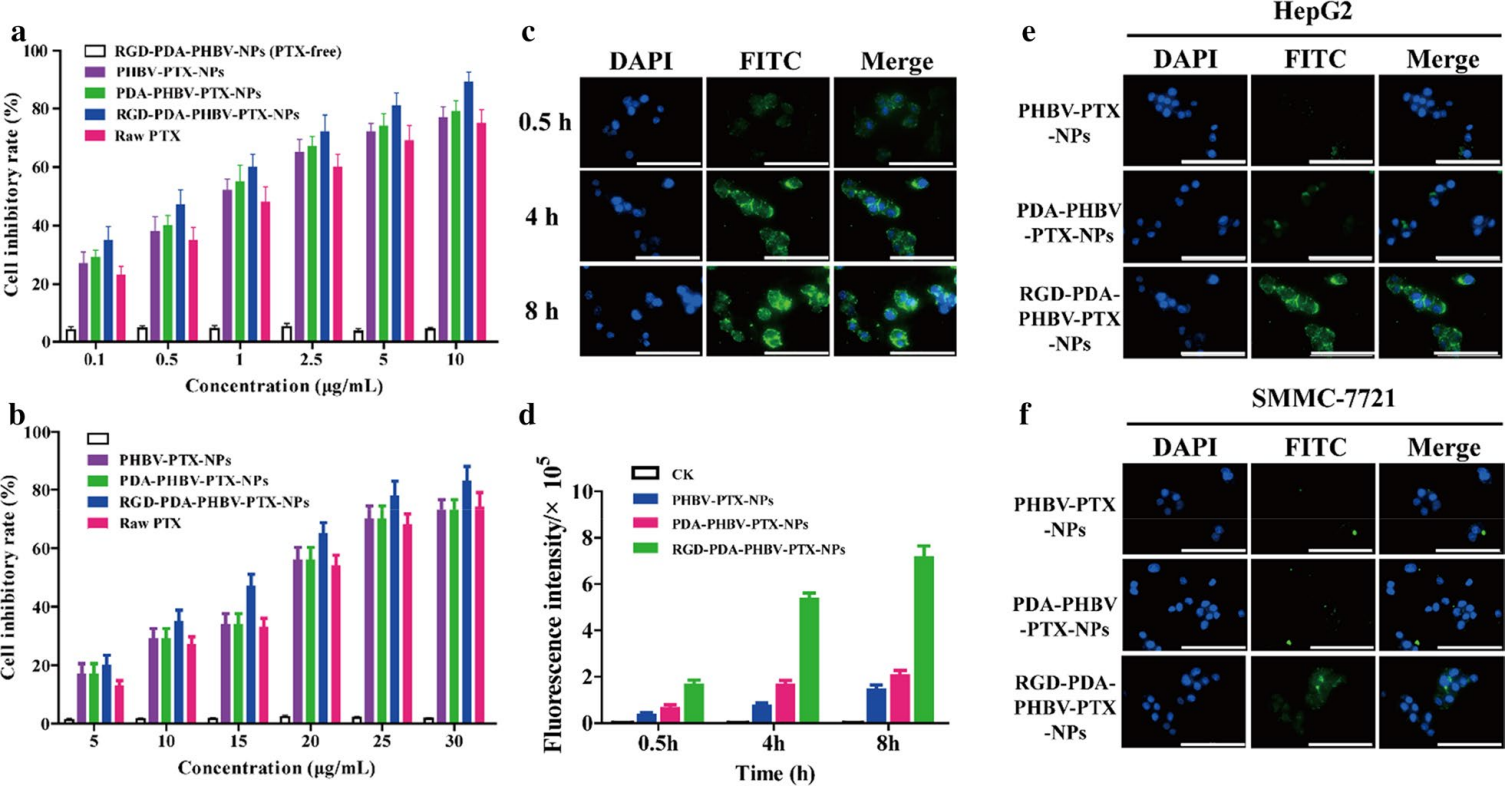
**a** The inhibitory effect of nanoparticles on HepG2 cells. **b** The inhibitory effect of nanoparticles on SMMC-7721 cells. **c** CLSM images of HepG2 cells incubated with RGD-PDA-PHBV-PTX-NPs for 0.5, 4 and 8 h. **d** The fluorescence intensity of HepG2 cells incubated with different nanoparticles for 0.5 h, 4 h and 8 h. CLSM images of HepG2 cells (**e**) and SMMC-7721 cells (**f**) incubated with different nanoparticles for 4 h. Scale bar: 50 μm. PHBV, ploy (3-hydroxybutyrate-co-3-hydroxyvalerate); PTX, paclitaxel; PDA, polydopamine; NPs, nanoparticles

The MTT assay was conducted to estimate the cytotoxicity of various PTX preparations. HepG2 and SMMC-7721 cells were treated with PHBV-PTX-NPs, PDA-PHBV-PTX-NPs, RGD-PDA-PHBV-PTX-NPs, raw PTX and RGD-PDA-PHBV-NPs (PTX-free) at different concentrations for 48 h. All PTX preparations reduced the viability of HCC cells in a dose-dependent manner with IC_50_ values of 0.78 ± 0.04, 0.89 ± 0.06, 1.08 ± 0.08, 1.23 ± 0.09 μg/mL in HepG2 cells, and 16.1 ± 0.97, 18.5 ± 1.06, 18.1 ± 0.94, 19.3 ± 1.09 μg/mL in SMMC-7721, respectively (Fig. 6a, b). As expected, the inhibitory effect of PTX NPs on HCC cell proliferation was stronger than that of raw PTX. It is mainly attributed to the prominent targeting effect of RGD, which can specifically recognize integrin αvβ3/αvβ5 on the surface of HCC cells [37]. More importantly, we did not observe an apparent cytotoxicity of drug-free NPs, suggesting that excipients (PHBV and PDA) are safe and highly biocompatible in cells.

Effective cell uptake behavior is an important index to evaluate the biological activity of chemotherapeutic agents [38]. Therefore, the uptake of RGD-PDA-PHBV-PTX-NPs in HepG2 cells was determined by the CLSM. As shown in Fig. 6c, the accumulation of RGD-PDA-PHBV-PTX-NPs at different time point was monitored in HepG2 cells. The strongest green fluorescence signal was observed in cytoplasm and nucleus at 8 h, and the fluorescence intensity was time-dependent. It is essential to transport PTX into the nucleus so that it can interact with genome DNA, and thereby exerting cytotoxicity [39]. In this study, the fluorescence intensity of HepG2 cells treated with RGD-PDA-PHBV-PTX-NPs for 4 h was significantly stronger than that of PDA-PHBV-PTX and PHBV-PTX-NPs (Fig. 6d). To further compare the uptake of RGD-PDA-PHBV-PTX-NPs, PDA-PHBV-PTX-NPs and PHBV-PTX-NPs in different cells, the NPs were incubated with HepG2 and SMMC-7721 cells for 4 h. As shown in Fig. 6e, f, the RGD-PDA-PHBV-PTX-NPs group showed the strongest green fluorescence in both HCC cells. It is noteworthy that we found the remarkably different fluorescence intensity between HepG2 and SMMC-7721 cells, which may be ascribed to differential expression of integrin on the cell surface.

### In vivo NIRF imaging of RGD-PDA-PHBV-PTX-NPs

**Fig. 7:**
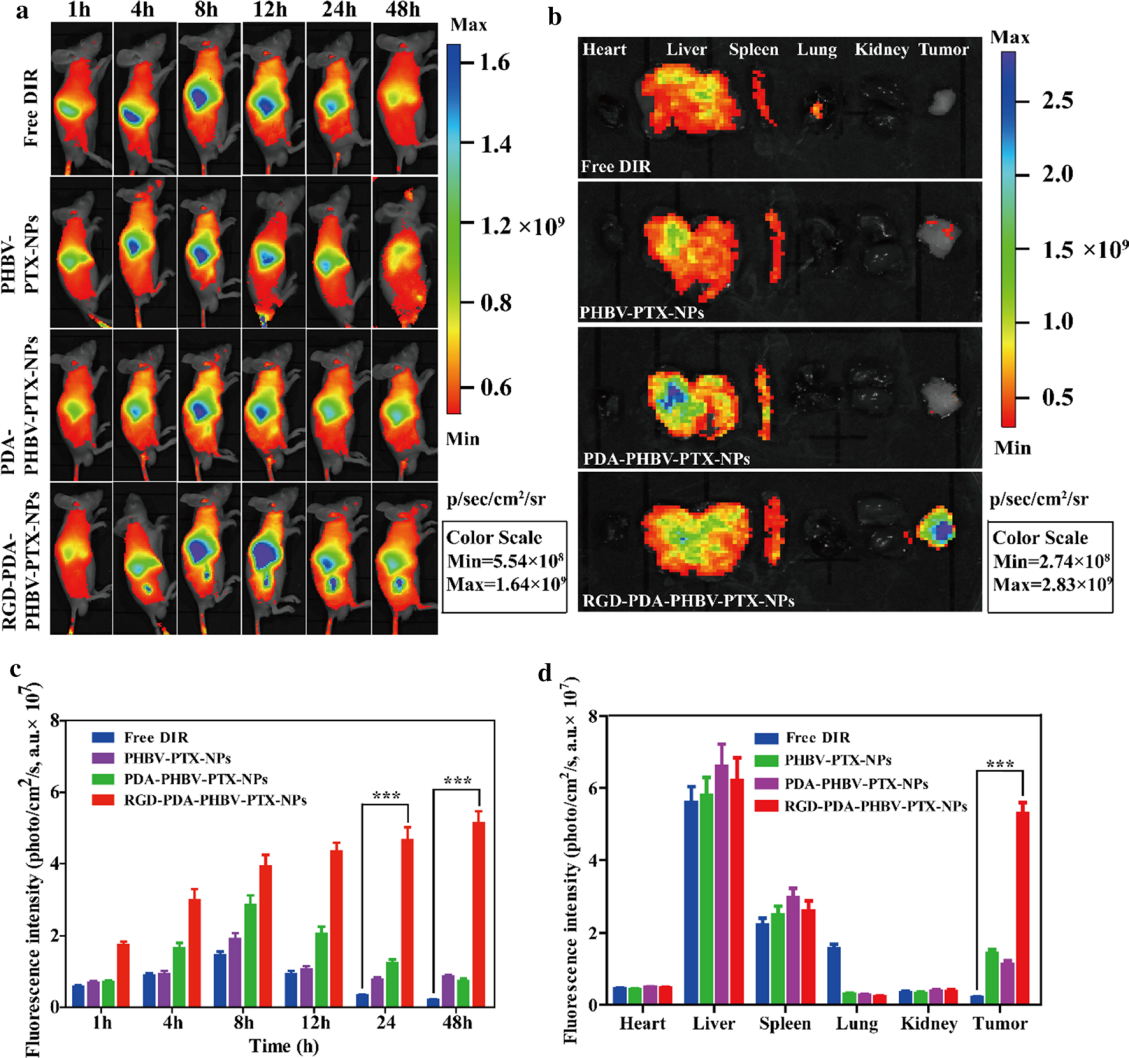
The distribution of free DIR, PHBV-PTX-NPs, PDA-PHBV-PTX-NPs and RGD-PDA-PHBV-PTX-NPs toward HepG2 tumor-bearing mice. **a** In vivo NIRF imaging in the tumor areas of the xenograft mouse model treated with free DIR, PHBV-PTX-NPs, PDA-PHBV-PTX-NPs and RGD-PDA-PHBV-PTX-NPs at 1, 4, 8, 12, 24 and 48 h tail vein injection. **b** In vivo fluorescence images of major organs of the xenograft mouse model at the end of the experiment. **c** Average NIRF intensity in the tumor areas. **d** Quantitative examination of nanoparticles in the tumor and main organs at 48 h tail vein injection, and each point represents the mean ± SD (n = 3). Student's t-test, *p < 0.05; **p < 0.01, ***p < 0.001. PHBV, ploy (3-hydroxybutyrate-co-3-hydroxyvalerate); PTX, paclitaxel; PDA, polydopamine; NPs, nanoparticles

To evaluate the targeting and in vivo distribution of free DIR, PHBV-PTX-NPs, PDA-PHBV-PTX-NPs and RGD-PDA-PHBV-PTX-NPs, near infrared fluorescence imaging was monitored at different time points after intravenous injection of drugs in tumor bearing nude mice. As shown in Fig. 7a, all the drugs were principally gathered in the liver within the first hour. 4 h post-injection, strong fluorescence signals were observed at the tumor site in the RGD-PDA-PHBV-PTX-NPs group. In contrast, it was found that the weak fluorescence signal was showed at the tumor tissue of mice with the free DIR, PHBV-PTX-NPs and PDA-PHBV-PTX-NPs. In addition, the strong fluorescence signal of RGD-PDA-PHBV-PTX-NPs could still be observed in tumor tissue 48 h after injection. However, there were very weak fluorescence signals in tumor tissues of mice in the free DIR group, PHBV-PTX-NPs group and PDA-PHBV-PTX-NPs group. These phenotypes demonstrated that the accumulation of RGD-PDA-PHBV-PTX-NPs in the tumor site was higher than that in the non-targeted group (free DIR, PHBV-PTX-NPs and PDA-PHBV-PTX-NPs), which may be due to the targeting mediated by ligand (RGD) and αvβ3/αvβ5 integrin. Additionally, to quantitatively analyze the tumor targeting capability of RGD-PDA-PHBV-PTX-NPs, we carried out fluorescence measurements (Fig. 7c). The result was well in agreement with the above phenotypes.

Ex vivo imaging could improve the sensitivity and reduce the background noise of light in the course of penetrating skin and tissue, so that the fluorescence signal can be detected more accurate. The fluorescence signals of the main organs (heart, liver, spleen, lung and kidney) and tumors of all groups of nude mice at 48 h after injection are shown in Fig. 7b. The quantitative fluorescence intensity was summarized in Fig. 7d. The results showed that fluorescence was mainly distributed in liver, spleen and tumor, secondly in lung, and least in heart and kidney. The accumulation of RGD-PDA-PHBV-PTX-NPs in the tumor site was the highest after, which indicated that RGD-PDA-PHBV-PTX-NPs had favorable targeting ability in vivo.

### In vivo antitumor efficacy

**Fig. 8:**
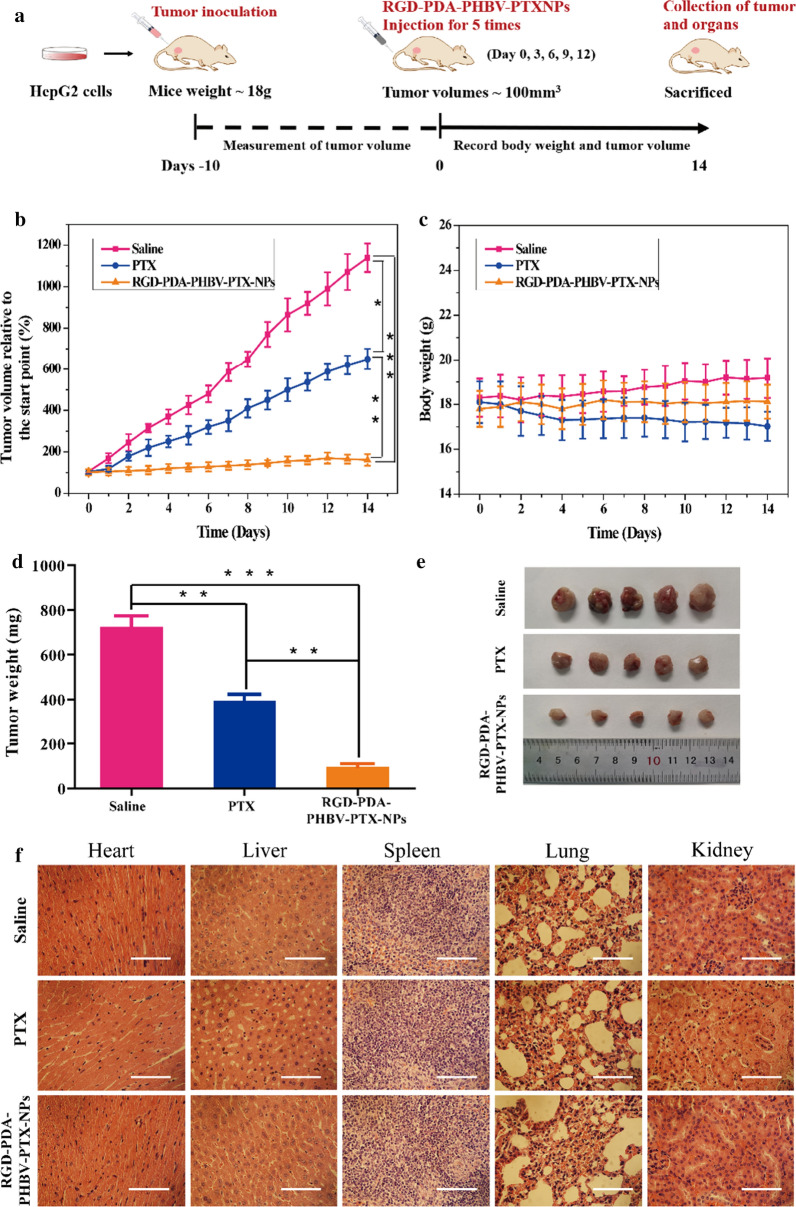
**a** Schematic illustration of the tumor suppression experiment. **b** The changes of tumor volumes curve. **c** Body weight of mice was measured during the 14 observation days in different groups. **d** Tumor weight of mice after treatment with saline, raw PTX and RGD-PDA-PHBV-PTX-NPs (data shown as means ± SD, n = 5, one way ANOVA, *p < 0.05, **p < 0.01, ***p < 0.001). **e** Images of excised tumors after treatment with different formulations. **f** H&E staining of tissues. Scale bar: 200 μm. PHBV, ploy (3-hydroxybutyrate-co-3-hydroxyvalerate); PTX, paclitaxel; PDA, polydopamine; NPs, nanoparticles

To evaluate the antitumor effect of RGD-PDA-PHBV-PTX-NPs in vivo, normal saline, raw PTX and RGD-PDA-PHBV-PTX-NPs were injected into the mice with HepG2 xenograft. For the dosage of free PTX, we referenced the published researches on PTX administration in vivo [40–42], and eventually chose intravenous injection with a 4 mg/kg of dosage in our study. Further, according to the PTX loading efficiency (14.21%), the concentration of RGD-PDA-PHBV-PTX-NPs was set as 28 mg/kg. Figure 8a showed a schematic diagram of the entire treatment process. The curve of tumor volume change is shown in Fig. 8b. During a 14-day treatment, the tumor volume in the saline group grew rapidly, while the antitumor effect of RGD-PDA-PHBV-PTX-NPs group was significant. The antitumor effect of raw PTX group is weaker than that of RGD-PDA-PHBV-PTX-NPs group, which may be due to the low tumor permeability, fast metabolism of raw PTX. The average tumor weight in the RGD-PDA-PHBV-PTX-NPs group was 96.65 ± 27 mg (Fig. 8d, e). It was significantly lower than that of in saline group and raw PTX group (723.58 ± 83 mg and 329.21 ± 44 mg, respectively, *p* < 0.05). Figure 8c showed the weight changes of the xenograft mouse model during treatment. There was no significant change in body weight in RGD-PDA-PHBV-PTX-NPs group, indicating that no obvious systemic toxicity in the course of treatment. In contrast, we observed that raw PTX group caused a declining trend of body weight. It indicates that the raw PTX administration leads to a certain toxicity on mice. Next, the mice were sacrificed after treatment, and the main organs (heart, liver, spleen, lung and kidney) were stained with hematoxylin and eosin (H&E). Compared with the saline group, the histomorphology of the main organs in the RGD-PDA-PHBV-PTX-NPs group was normal (Fig. 8f). These results suggested that targeted chemotherapy based on RGD-PDA-PHBV-PTX-NPs was safe and less toxic, which could provide the possibility for clinical application in the future.

## Conclusion

In summary, we have successfully developed a biodegradable RGD-PDA-PHBV-PTX-NPs for precise treatment of HCC. In this nanosystem, PTX was used as the chemotherapeutic agent. PDA was served as a switch for the pH-responsive release, which can greatly improve drug stability and avoid premature release. The RGD-PDA-PHBV-PTX-NPs, which were produced by optimizing preparation conditions, showed multiple advantages including small particle size, high drug loading efficiency, favorable biocompatibility, rich accumulation in tumor tissue, and intelligent stimulation release. It is able to effectively target HCC cells through the specific recognition of αvβ3/αvβ5 integrin. Additionally, the slow release of PTX and use of PHBV significantly reduce toxicity and side effects for normal organs. More importantly, the RGD-PDA-PHBV-PTX-NPs not only has excellent stability in aqueous solution, but also improve the solubility of PTX. The in vitro and in vivo test confirmed that RGD-PDA-PHBV-PTX-NPs had enhanced therapeutic and antitumor effect compared to their counterparts. In conclusion, our data suggested that functional NPs based on PHBV can be used as a potential carrier for future HCC therapy.

## Supplementary Information

**Additional file 1: Table S1. **Summary of reports available on production of paclitaxel particles.** Figure S1.** Effect of PTX concentration on drug loading efficiency.
