# Supplementary material for: pH-responsive delivery vehicle based on RGD-modified polydopamine-paclitaxel-loaded poly (3-hydroxybutyrate-co-3-hydroxyvalerate) nanoparticles for targeted therapy in hepatocellular carcinoma
Source: J Nanobiotechnology. 2021 Feb 6;19:39. doi: 10.1186/s12951-021-00783-x (PMC7866683; doi:10.1186/s12951-021-00783-x)
Supplement: Supplementary file 1 — Additional file 1: Table S1. Summary of reports available on production of paclitaxel particles. Figure S1. Effect of PTX concentration on drug loading efficiency. [file 12951_2021_783_MOESM1_ESM.docx]

Additional file 1

**Title:** pH-Responsive Delivery Vehicle Based on RGD-Modified Polydopamine-Paclitaxel-Loaded Poly (3-hydroxybutyrate-co-3-hydroxyvalerate) Nanoparticles for Targeted Therapy in Hepatocellular Carcinoma

Mingfang Wu^1,2#^, Chen Zhong^4#^, Qian Zhang^1,3^, Lu Wang^1,3^, Lingling Wang^1,3^, Yanjie Liu^1,3^, Xiaoxue Zhang^1,3^, Xiuhua Zhao^1,3^*

1. College of Chemistry, Chemical Engineering and Resource Utilization, Northeast Forestry University, Harbin 150040, Heilongjiang, China;
2. School of Biological and Chemical Engineering, Zhejiang University of Science and Technology, Hangzhou, 310023, Zhejiang, China;
3. Key Laboratory of Forest Plant Ecology, Northeast Forestry University, Ministry of Education, Harbin 150040, Heilongjiang, China;
4. State Key laboratory of Genetic Engineering, School of Life Sciences, Fudan University, Shanghai 200438, China

^#^ Mingfang Wu and Chen Zhong contributed equally to this work.

* Correspondence: Xiuhua Zhao, College of Chemistry, Chemical Engineering and Resource Utilization, Northeast Forestry University, 26 hexing road, harbin 150040, heilongjiang, china; Tel +86 451 8219 1517; Fax +86 451 8210 2082; E-mail: [xiuhuazhao@nefu.edu.cn](mailto:xiuhuazhao@nefu.edu.cn)

Table S1. Summary of reports available on production of paclitaxel particles.

| Samples | Particle size obtained (nm) | Polydispersity index (PDI) | Drug loading efficiency  (%) | Entrapment efficiency (%) | Cumulative release  (%) pH=7.4 | Reference |
| --- | --- | --- | --- | --- | --- | --- |
| PLGA-PTS NPs | 200±27 | 0.04 | 0.7 | 85 | 60.00±2.73 | Sepideh Khodaverdi et al., 2020 |
| PLGA-PVA-PTX NP1 | 223±6 | 0.02 | 8.5 | 92 | 88.3±2.45 | Fatemeh Madani et al., 2020 |
| PLGA-PVA/P188-PTX NP2 | 241±19 | 0.07 | 8.4 | 89 | 79.1±3.21 | Fatemeh Madani et al., 2020 |
| DTX-loaded pD-TPGS-PLA | 205.2±8.3 | 0.137 | 8.07 | 89.25 | 42.3±5.02 | Dunwan Zhu et al., 2016 |
| DTX-loaded Gal- pD-TPGS-PLA | 209.4±5.1 | 0.145 | 7.69 | 88.47 | 40.3±5.15 | Dunwan Zhu et al., 2020 |
| Paclitaxel-loaded M-PLA-TPGS NPs | 123±2.9 | 0.159 | 10 | 98 | 48.3±1.62 | Kebing Wang et al., 2014 |
| PTX-NPs (PVP-b-PCL as drug carriers) | 115.5±13.3 | — | 17.2 | 91.2 | 69.3±3.21 | Donghui Zheng et al., 2015 |
| PTX-loaded CS-g-PCL | 529 ± 33 | 0.451 | 33.3 | 75.3 | — | Andreia Almeida et al., 2017 |

Abbreviation: PLGA, polylactic glycolic acid; PVA, polyvinyl alcohol; PLA, Poly(lactide); NP, nanoparticle; TPGS, a water-soluble derivative of natural vitamin E; Gal, galactosamine; PCL, poly(-caprolactone); PHBV, ploy (3-hydroxybutyrate-co-3-hydroxyvalerate); DTX, docetaxel.

**
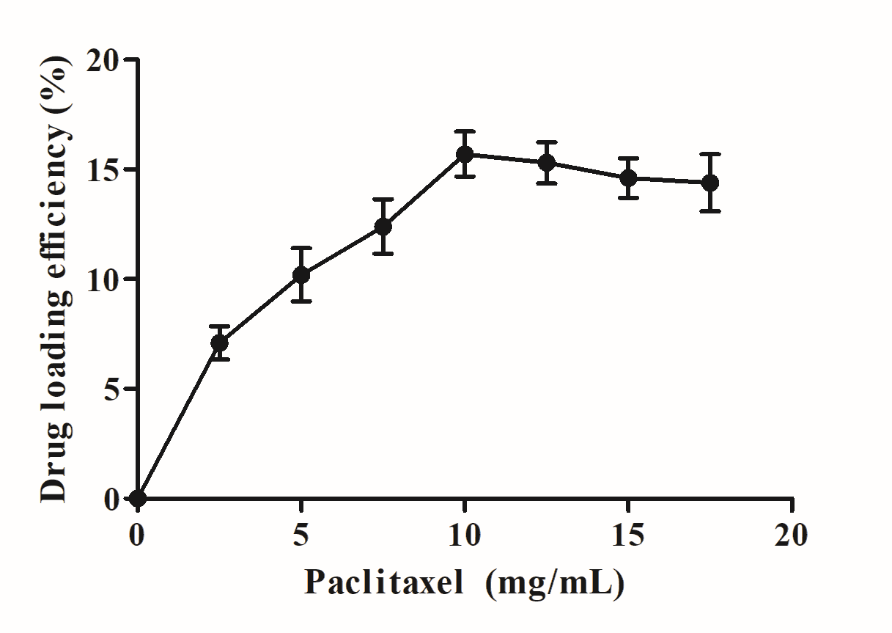
**

Figure.S1 Effect of PTX concentration on drug loading efficiency
